# Synergic effects between ocellatin-F1 and bufotenine on the inhibition of BHK-21 cellular infection by the rabies virus

**DOI:** 10.1186/s40409-015-0048-1

**Published:** 2015-12-02

**Authors:** Rene dos Santos Cunha Neto, Hugo Vigerelli, Carlos Jared, Marta Maria Antoniazzi, Luciana Botelho Chaves, Andréa de Cássia Rodrigues da Silva, Robson Lopes de Melo, Juliana Mozer Sciani, Daniel C. Pimenta

**Affiliations:** Butantan Institute, Laboratory of Biochemistry and Biophysics, Av. Vital Brazil, 1500, São Paulo, SP 05503-900 Brazil; Pasteur Institute, Laboratory of Rabies Diagnostic, Serology, Avenida Paulista, 393, São Paulo, 01311-000 SP Brazil; Butantan Institute, Laboratory of Cell Biology, Av Vital Brasil, 1500, São Paulo, 05503-900 SP Brazil; Butantan Institute, Special Laboratory of Toxinology, Av Vital Brasil, 1500, São Paulo, 05503-900 SP Brazil

**Keywords:** Rabies virus, Ocellatin-F1, Bufotenine, Toxin, *Leptodactylus*

## Abstract

**Background:**

Rabies is an incurable neglected zoonosis with worldwide distribution characterized as a lethal progressive acute encephalitis caused by a lyssavirus. Animal venoms and secretions have long been studied as new bioactive molecular sources, presenting a wide spectrum of biological effects, including new antiviral agents. Bufotenine, for instance, is an alkaloid isolated from the skin secretion of the anuran *Rhinella jimi* that inhibits cellular penetration by the rabies virus. Antimicrobial peptides, such as ocellatin-P1 and ocellatin-F1, are present in the skin secretion of anurans from the genus *Leptodactylus* and provide chemical defense against predators and microorganisms.

**Methods:**

Skin secretion from captive *Leptodactylus labyrinthicus* was collected by mechanical stimulation, analyzed by liquid chromatography and mass spectrometry, and assayed for antiviral and cytotoxic activities. Synthetic peptides were obtained using solid phase peptide synthesis, purified by liquid chromatography and structurally characterized by mass spectrometry, and assayed in the same models. Cytotoxicity assays based on changes in cellular morphology were performed using baby hamster kidney (BHK-21) cells. Fixed *Rabies virus* (Pasteur Virus – PV) strain was used for virological assays based on rapid fluorescent focus inhibition test.

**Results:**

Herein, we describe a synergic effect between ocellatin-F1 and bufotenine. This synergism was observed when screening the *L. labyrinthicus* skin secretion for antiviral activities. The active fraction major component was the antimicrobial peptide ocellatin-F1. Nevertheless, when the pure synthetic peptide was assayed, little antiviral activity was detectable. In-depth analyses of the active fraction revealed the presence of residual alkaloids together with ocellatin-F1. By adding sub-effective doses (e.g. < IC_50_) of pure bufotenine to synthetic ocellatin-F1, the antiviral effect was regained. Moreover, a tetrapetide derived from ocellatin-F1, based on alignment with the virus’s glycoprotein region inferred as a possible cell ligand, was able to maintain the synergic antiviral activity displayed by the full peptide.

**Conclusions:**

This novel antiviral synergic effect between a peptide and an alkaloid may present an innovative lead for the study of new antiviral drugs.

**Electronic supplementary material:**

The online version of this article (doi:10.1186/s40409-015-0048-1) contains supplementary material, which is available to authorized users.

## Background

Rabies is an incurable zoonosis with worldwide distribution characterized as a lethal progressive acute encephalitis responsible for more than 55 thousand deaths per year, mostly in Asia and Africa [1]. In Brazil, according to the Ministry of Health, there have been 780 notified human rabies cases from 1986 to 2015 [2]. It is caused by a neurotropic enveloped virus, the *Rabies virus*, which has a single non-segmented negative strand RNA genome that encodes five proteins: nucleoprotein, RNA-dependent RNA polymerase, phosphoprotein, matrix protein and glycoprotein [3].

Glycoprotein is a membrane protein that forms trimers on the viral surface and it is the major antigen responsible for inducing the production of virus-neutralizing and protective antibodies [4]. It is also the protein that most contributes to the viral pathogenicity, by binding to specific host cell receptors, promoting the virus’s entry into the cells [5]. The first described target for glycoprotein binding was a nicotinic acetylcholine receptor (nAChR) [6]. Other molecules, such as the neural cell adhesion molecule (NCAM) and the low-affinity neurotrophin receptor (p75NTR), are also considered mediators of the virus’s entrance into the host cells [7, 8].

Different organisms have been used as sources of biologically active molecules [9]. Amphibian skin extract and secretions contain a great diversity of bioactive compounds that provide chemical protection against predators and microorganisms [10]. Several bioactive molecules, such as alkaloids, steroids and peptides, have been isolated from the anurans skin secretion [11–13]. The genus *Leptodactylus*, which includes 75 species spread across southern North America, South America and the West Indies, has already been prospected for such bioactive molecules, including by our group [14, 15]. The alkaloid leptodactyline, for example, possesses muscle relaxant action and neuromuscular blocking effects [16]. Ocellatin-P1 (formerly known as pentadactylin, according to the nomenclature proposed by Conlon [17]), an antimicrobial peptide isolated from the skin secretion of *Leptodactylus pentadactylus*, is active against several gram-negative and gram-positive bacteria; whereas ocellatin-F1 (previously known as fallaxin), obtained from the giant ditch frog *Leptodactylus fallax* skin secretion, presents antimicrobial activity against gram-negative bacteria [17–19].

Taking into account that the amphibian skin secretions are rich sources of bioactive molecules – including antiviral molecules – and that the biochemical characterization of molecules isolated from *Leptodactylus labyrinthicus* is still incipient, the aim of this study was to screen the skin secretion of *L. labyrinthicus* to search for bioactive molecules that actively inhibit infection by the rabies virus in BHK-21 cell line.

## Methods

### Reagents

All reagents employed in this work were of analytical grade and were purchased from Sigma Aldrich Co. (USA), unless otherwise stated. Bufotenine was purified from *Anadenanthera colubrina* seeds, as previously described [11].

### Collection of specimens and extraction of skin secretions

The collection and housing of *L. labyrinthicus* were performed under license number 15964–1 from the Brazilian Institute of Environment and Renewable Natural Resources (IBAMA). Three adult male specimens were collected in Araraquara (SP, Brazil) and maintained in captivity at the Laboratory of Cell Biology, Butantan Institute (SP, Brazil). The skin secretions were mechanically collected by submerging and gently compressing the specimens in deionized water. The aqueous solutions obtained were lyophilized and stored at −20 °C.

### Samples preparation

Skin secretions were filtered in 10 kDa cut-off membranes (Amicon Ultra-4, Millipore). Filtered contents were used in the subsequent steps.

### Reversed-phase liquid chromatography

Skin secretion solutions were analyzed by reversed-phase high-performance liquid chromatography (RP-HPLC) using a binary HPLC system (20A Prominence, Shimadzu Co., Japan). Aliquots of the secretions were loaded in a C18 monolithic column (Phenomenex, 100 mm × 4.6 mm), in a two solvent system: (A_1_) trifluoroacetic acid (TFA)/water (1:1,000) and (B_1_) TFA/acetonitrile (ACN)/water (1:900:100). The column was eluted at a constant flow rate of 2 mL.min^−1^ with a 0 to 100 % gradient of solvent B_1_ over 13 min. The eluates were monitored by a Shimadzu SPD-M20A detector at 214 nm. After skin secretion profiles, fractions were manually collected and dried for subsequent assays.

### Mass spectrometry

#### Liquid chromatography-mass spectrometry (LC-MS)

LC-MS analyses were performed using an electrospray-ion trap-time of flight (ESI-IT-TOF) (Shymadzu Co., Japan) mass spectrometer equipped with binary ultra-fast liquid chromatography system (UFLC) (20A Prominence, Shimadzu Co., Japan). Aliquots of each material were dried, resuspended in 0.1 % formic acid (FA) and loaded in a C18 column (Shimadzu-pack XR-ODS, 2.2 μm; 100 mm × 3 mm) in a binary solvent system: (A_2_) FA/water (1:1,000) and (B_2_) FA/ACN/water (1:900:100). The column was eluted at a constant flow rate of 0.2 mL.min^−1^ with a 0 to 100 % gradient of solvent B_2_ over 20 min. The eluates were monitored by a Shimadzu SPD-M20A PDA detector before introduction into the mass spectrometer, in which the spray voltage was kept at 4.5 kV, the capillary voltage at 1.76 kV, and temperature at 200 °C. MS spectra were acquired under positive ionization mode and collected in the 80–2,000 *m*/*z* range. Instrument control, data acquisition, and data processing were performed with LabSolutions (LCMS solution 3.60.361 version, Shimadzu Co., Japan).

#### Direct infusion mass spectrometry

MS analyses were performed in an ESI-IT-TOF as described above. Previously dried skin secretion fractions were resuspended in 0.5 % FA, for positive mode electrospray ionization (ESI+), and manually injected in a Rheodyne injector at a flow rate of 50 μL.min^−1^, in 50 % FA/ACN/water (5:900:100). Instrument control, data acquisition, and data processing were performed by the software LabSolutions (LCMS solution 3.60.361 version, Shimadzu Co., Japan).

#### Peptide synthesis

Synthetic peptides were obtained in an automated bench-top simultaneous multiple solid-phase synthesizer (PSSM 8 system from Shimadzu Co., Japan) using solid-phase peptide synthesis by the Fmoc-Procedure [20, 21]. Peptides were purified by reversed-phase chromatography (Shimadzu Co., Shim-pack Prep-ODS, 5 μm, 20 × 250 mm) semi-preparative HPLC, whereas the purity and identity of the peptides were confirmed by ESI-IT-TOF mass spectrometry and by analytical HPLC, under the same conditions described above.

### Cell culture and viruses

Baby hamster kidney (BHK-21, ATCC CCL-10) cells, grown in Eagle’s minimum essential medium (MEM), containing Earle’s salts supplemented with 10 % fetal bovine serum (FBS), were used for the cytotoxicity and virology assays. The fixed *Rabies virus* (PV) strain was used for the virology assay [22]. The assays were performed in 96-well microplates.

### Cytotoxicity assay

The cytotoxic effect of different molecules was evaluated by a test based on morphological changes of BHK-21 cells.

Briefly, samples were diluted in MEM-FBS and deposited in 96-well microplates, according to the items described below. Using MEM-FBS as diluent, serial fourfold dilutions of these samples were performed in 50 μL volumes. Then, 100 μL of BHK-21 cells suspension (2.5 × 10^5^ cells.mL^−1^) were added to each well and incubated at 37 °C for 20 h, in a 5 % CO_2_ atmosphere. Following incubation, cells were observed in an optical microscope (Carl-Zeiss), using 100× magnification; formation of a confluent cell monolayer was analyzed in comparison to positive [dimethyl sulfoxide – DMSO diluted into MEM-FBS (1:5)] and negative (MEM-FBS) cytotoxicity controls.

### Virology assay

The effect of different molecules, dissolved into MEM-FBS, as interfering agents of rabies virus infection in BHK-21 cells, was evaluated by rapid fluorescent focus inhibition test (RFFIT) adapted for microplates [23, 24]. Samples were added to 96-well microplates, according to the description below.

Using MEM-FBS as diluent, serial fourfold dilutions of these samples were performed in 50 μL volumes. PV strain suspension previously titered (30 FFD_50_), 50 μL, was added to each well. Then, 100 μL of BHK-21 cellular suspension (2.5 × 10^5^ cells.mL^−1^) was added per well and microplates were incubated at 37 °C, for 20 h, in a 5 % CO_2_ atmosphere. After incubation, the medium was aspirated and microplates were cooled on ice. Cell monolayer was fixed by adding cold 80 % acetone into the wells. After 15 min, acetone was discarded by inversion and microplates were dried at 37 °C. The staining step was performed by adding 40 μL of antirabies immunoglobulin conjugated with fluorescein isothiocyanate produced by Instituto Pasteur (SP, Brazil) [25], previously titrated. Microplates were incubated at 37 ° C, for 1 h, in a 5 % CO_2_ atmosphere. After this, microplates were washed by submerging them in three changes of phosphate buffered saline (PBS) and then in three changes of distilled water. Microplates were inverted and shaken for water removal. Finally, 50 μL of glycerin/PBS solution (1:10) was added to each well. BHK-21 cells + MEM-FBS and PV + BHK-21 cells + MEM-FBS were used, respectively, as cell viability and virus infectivity controls. Cells were observed in an inverted fluorescence microscope (Leica DMIL), using 200× magnification, and 18 fields per well were analyzed, and fields containing fluorescent foci (infected cells) were counted.

### Statistical analyses

Samples were assayed in duplicates in both assays. Results are presented as mean ± standard error of mean. Statistical evaluation of data was carried out by one-way analysis of variance (ANOVA) followed by Tukey post-test (virology assay). Differences were considered statistically significant when *p* < 0.05.

## Results

**Fig. 1:**
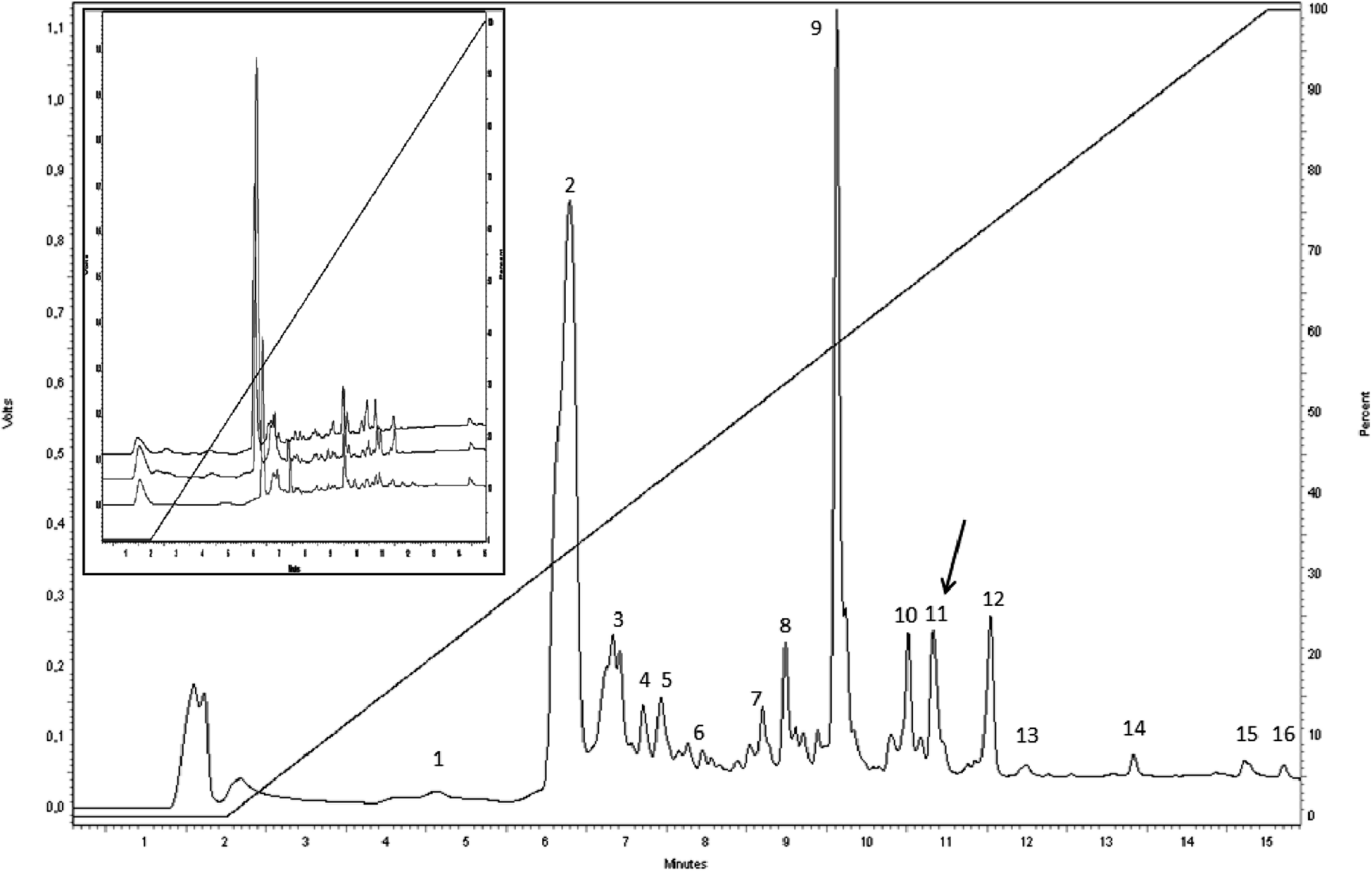
Representative RP-HPLC profile of the pooled filtered skin secretion of *L. labyrinthicus.* RP-HPLC profile of *L. labyrinthicus* skin secretion in a C18 monolithic column monitored at 214 nm. Numbers 1–16 represent the sixteen fractions manually collected for the antiviral screening; the arrow indicates the active fraction. Inset: superimposed individual chromatographic profiles of the filtered skin secretions from three individuals, under the same chromatographic conditions

Initial RP-HPLC analyses of the crude *L. labyrinthicus* skin secretions (Fig. 1, inset) suggested similar profiles among the three specimens, allowing the pooling of the individual secretions for subsequent fractionation (Fig. 1). Fractions were manually collected, lyophilized and tested for cytotoxic and antiviral activity (data not shown). Under our experimental conditions only one fraction (F11) was active and is indicated by the arrow, in Fig. 1.

**Fig. 2:**
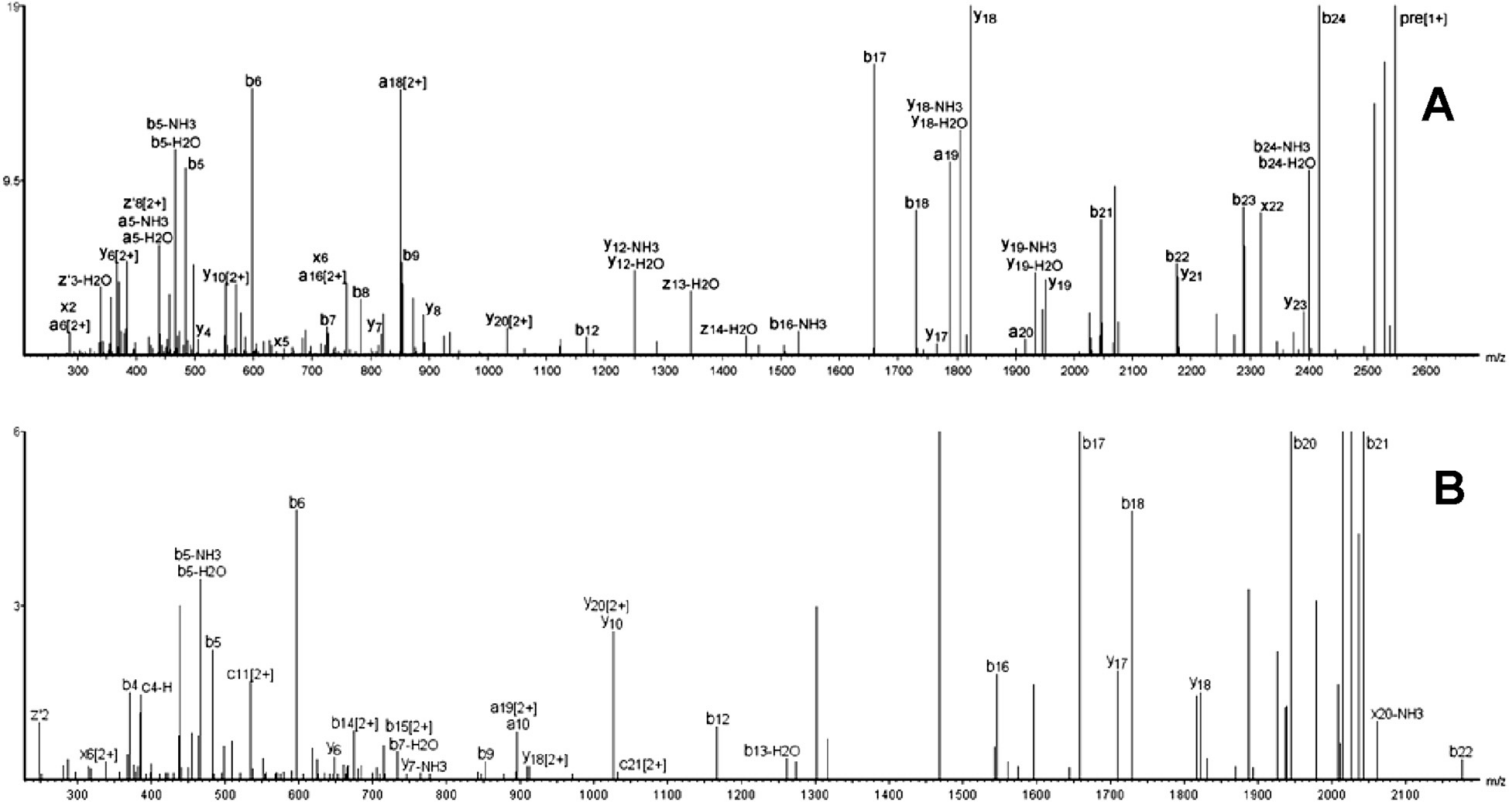
Representative deconvoluted annotated *de novo* MS^2^ spectra of ocellatin-F1 and Des-Lys^24^-Leu^25^-ocelatin-F1. **a** ocellatin-F1 and **b** Des-Lys^24^-Leu^25^-ocelatin-F1, as analyzed by Peaks Studio v7

Mass spectrometric analyses of F11 (Additional file 1) showed the presence of two peptides: (2547.98 ± 0.16 Da and 2192.80 ± 0.16 Da), as well as other minor low-molecular-mass compounds. Both peptides were selected for MS^2^ CID fragmentation (Additional file 2, MS^2^ of m/z = 850.16) and were *de novo* sequenced with the aid of the software Peaks Studio v7 (Bionformatics Solutions Inc., Canada), being identified as ocellatin-F1 (GVVDILKGAAKDIAGHLASKVMNKL; Fig. 2a) and Des-Lys^24^-Leu^25^-ocellatin-F1 (GVVDILKGAAKDIAGHLASKVMN; Fig. 2b). Ocellatin-F1, termed OF1, was then synthesized by the Fmoc procedure and the MS^2^ CID spectrum of the triply charged peptide is presented in Additional file 2, for the sake of comparison with the natural peptide fragmentation pattern of the corresponding ion.

The peptide sequence of OF1 was then compared with the rabies virus glycoprotein region that was supposed to mediate virus internalization (Table 1) and similarity could be observed (boxed region) between the tetrapeptide associated with the nAChR recognition, N^194^-S-R-G^197^, and A^18^-S-K-V^21^ OF1 internal peptide. Both tetrapeptides were synthesized by the Fmoc strategy and assayed for antiviral activity under the same experimental conditions employed for the screening of the antiviral activity (Additional file 3). OF1 tetrapeptide was termed OF1TP and the rabies virus glycoprotein G tetrapeptide was termed RVGTP. At the tested concentrations, F11, synthetic OF1, OF1TP and RVGTP did not present cytotoxic activity (Additional file 4).

**Table 1:** Sequence alignment between ocellatin-F1 and the RABV glicoprotein G region associated with cell penetration

	

Only the boxed tetrapeptides were aligned; the gray region was not considered for the alignment. ^a^Uniprot: Q85422

**Fig. 3:**
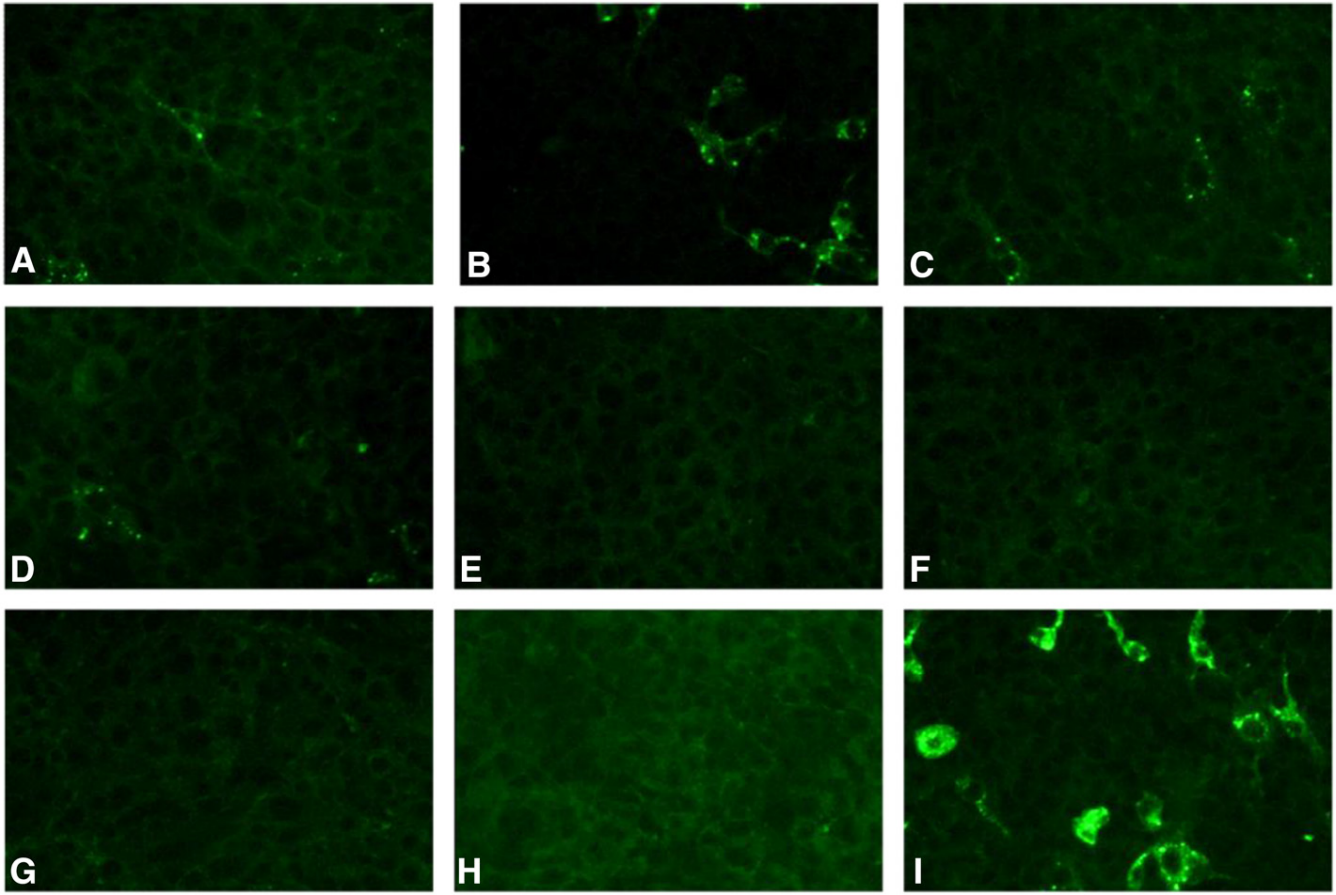
Direct immunofluorescence (DIF) of BHK-21 cell monolayer after treatment with RABV and different molecules. **a** F11 (1.0 mg.mL^−1^); **b** synthetic OF1 (1.0 mg.mL^−1^); **c** OF1TP (6.0 mg.mL^−1^); **d** bufotenine (0.8 mg.mL^−1^); **e** bufotenine (0.8 mg.mL^−1^) and F11 (1.0 mg.mL^−1^); **f** bufotenine (0.8 mg.mL^−1^) and synthetic OF1 (1.0 mg.mL^−1^); **g** bufotenine (0.8 mg.mL^−1^) and OF1TP (6.0 mg.mL^−1^); **h** negative control (MEM-FBS only) and **i** positive control (PV only). Magnification 200×

**Fig. 4:**
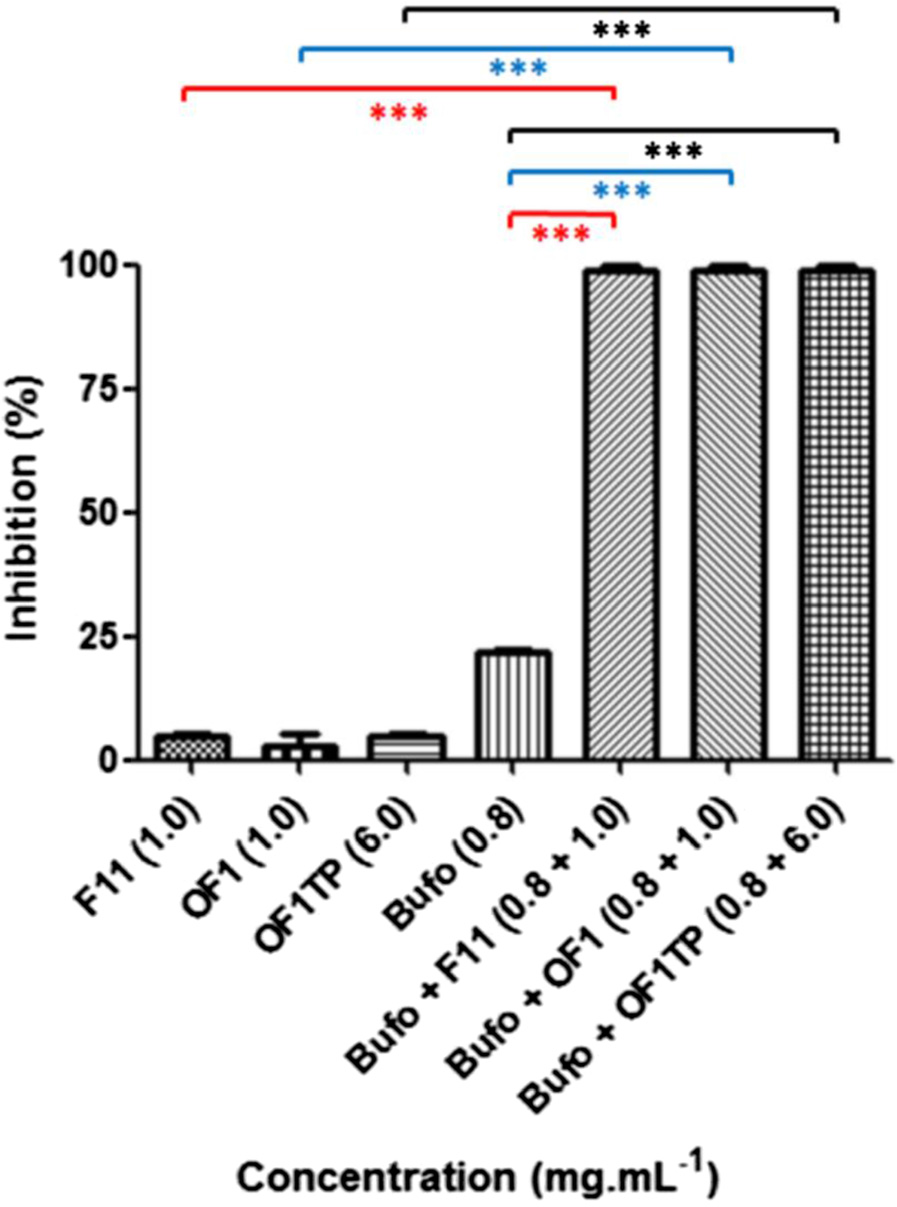
Effect of different molecules tested individually or in association as interfering agents of PV strain infection in BHK-21 cell line. Percentage of inhibition. The effect of active fraction and bufotenine tested individually compared to their effect in association (in red), the effect of synthetic ocellatin-F1 and bufotenine tested individually compared to their effect in association (blue), and the effect of synthetic ocellatin-F1 tetrapeptide and bufotenine tested individually compared to their effect in association (in black). F11: active fraction of *L. labyrinthicus* skin secretion; OF1: synthetic ocellatin-F1; OF1TP: synthetic ocellatin-F1 tetrapeptide; Bufo: bufotenine. ***(*p* < 0.001)

Figure 3 displays the direct immunofluorescence assays (DIF) used for the determination of the antiviral activity, assessed by means of identification and counting of viral particles inside the cells, revealed by the conjugated fluorescent antibody. These data were then converted into percentage of viral infection inhibition and are presented in Fig. 4. The F11 (1.0 mg.mL^−1^) was able to reduce by 6 % the viral infection (Figs. 3a and 4). However, when synthetic 1.0 mg.mL^−1^ OF1 was assayed under the same conditions, it caused a diminished inhibitory effect: 3 % inhibition of infection (Figs. 3b and 4). OF1TP was able to inhibit 6 % of the viral infection, but only when assayed at 6.0 mg.mL^−1^ (Figs. 3c and 4). On the other hand, RVGTP did not inhibit the viral infection (data not shown).

In order to understand the reduced effect of OF1 in relation to F11, its mass spectrum (Additional file 1) was re-analyzed, thereby revealing the presence of low-molecular-mass compounds, particularly m/z = 219.13, identified as N’,N’,N’-trimethyl-5-hydroxytryptamine (5-HTQ), based on the high accuracy of mass determination, which allowed formula prediction, and the presence of the characteristic spontaneous fragment m/z = 160.06. 5-HTQ is an alkaloid commonly found in amphibian skin secretions, often together with the antiviral alkaloid bufotenine. In order to investigate the possible synergic effect between alkaloids and OF1 on the inhibition of the RABV infection, sub-effective doses of pure plant-derived bufotenine were assayed under the same conditions (Fig. 3d) and in association with F11 (Fig. 3e), synthetic OF1 (Fig. 3f), OF1TP (Fig. 3g) and RVGTP (data not shown).

The 0.8 mg.mL^−1^ bufotenine dose (corresponding to half of the IC_50_ dose for the antiviral effect) was considered sub-effective, e.g., only capable of inducing mild effects and not 100 % inhibition [11]. This dose was able to cause a 22 % reduction in the viral infection (Fig. 4) and was not cytotoxic for BHK-21 cells (Additional file 4). On the other hand, when 0.8 mg.mL^−1^ bufotenine was added to 1 mg.mL^−1^ F11, 1 mg.mL^−1^ synthetic OF1 or 6 mg.mL^−1^ OF1TP, 100 % virus infection inhibition could be observed, indicating a synergic effect between the peptide and the alkaloid (Fig. 4), which is statistically different (*p* < 0.001) from the infection inhibitory effect of the molecules tested individually.

## Discussion

In this work, we report not only the isolation and biochemical characterization of ocellatin-F1 (OF1) and Des-Lys^24^-Leu^25^-OF1 from the skin secretion of *L. labyrinthicus*, but also that OF1 possesses mild antiviral effect on the inhibition of rabies virus infection in BHK-21 cells. This effect can reach 100 % when in synergism with bufotenine, an already know antiviral alkaloid isolated from the skin secretion of *Rhinella jimi* [11].

In this study, OF1 was isolated through a biological driven-assay, monitoring anti-rabies activity. The active fraction – which contained OF1, Des-Lys^24^-Leu^25^-OF1 and low-molecular-mass molecules – was able to decrease the PV rabies virus strain infection in BHK-21 cells.

OF1 was first isolated from the skin secretion of *L. fallax*, but has also been found in other species from the genus *Leptodactylus*, including *L. pentadactylus* and *L. labyrinthicus* [18, 19, 26]. It is a 25-amino-acid residue peptide able to inhibit the growth of gram-negative bacteria (*Escherichia coli, Pseudomonas aeruginosa, Enterobacter cloacae, Klebsiella pneumoniae*), but inactive against *Staphylococcus aureus* (gram-positive bacterium) and the yeast *Candida albicans* [19]. Rollins-Smith et al. [19] also mention the structural similarity between OF1 and ranatuerin-2, an antimicrobial peptide from *Rana catesbeiana*, which presents antimicrobial activity towards *S. aureus* [27]. Remarkably, antiviral activity of ranatuerin-2 has already been reported against the frog virus 3 (FV3) – capable of causing *Amphibia* mass mortality – and the channel catfish virus (CCV) – which causes significant economic loss in channel catfish farms [28]. Based on these findings, OF1 was elected as the candidate for the following antiviral tests, performed with the synthetic molecule. Nevertheless, pure synthetic OF1 was not as effective as F11 (Fig. 4).

Two possibilities may account for the reduced activity of OF1: ranatuerin has a disulfide bond at the C-terminus (between Cys^23^ and Cys^28^), whereas OF1 is a linear peptide; and the identity between OF1 and ranatuerin encompasses the central part of the peptide (^6^LKGAAKDI^13^AGH^16^L^17^), with Ile^13^ and His^16^ being not identical but conserved. Moreover, this identity ceases before the ring region in ranatuerin and at the residue (Leu^17^) immediately prior to the tetrapeptide considered similar to the rabies virus glycoprotein region associated with the viral cell penetration (Table 1). Therefore, the determinants of specificity for the antiviral activity appear to be located at the C-terminal of these peptides and are distinct.

To test this hypothesis, a fragment of the C-terminal region of OF1 (considered similar to the rabies virus glycoprotein) was synthesized and assessed for antiviral activity. Although the dose was higher (6 mg.mL^−1^ versus 1 mg.mL^−1^ for OF1), OF1TP was able to retain its antiviral activity, corroborating the hypothesis that the antiviral activity of these antimicrobial peptides is located at their C-termini.

In-depth analyses of the mass spectra of the active fraction from *L. labyrinthicus* skin secretion (F11, Additional file 1) showed that, besides Des-Lys^24^-Leu^25^-OF1 that also contains the OF1TP sequence, low-molecular-mass molecules, particularly 5-HTQ, were also present. Our group has recently described the antiviral effect of bufotenine, structurally a closely related 5-HTQ alkaloid. Bufotenine is an alkaloid with noxious properties which works as a defense mechanism against predators and is able to inhibit 100 % of rabies infection in BHK-21 cells, at 3.9 mg.mL^−1^ [10, 11, 29].

Based on these pieces of information, a possible synergic effect between bufotenine and OF1 was assayed (Fig. 3e, f and g and Fig. 4). The level 0.8 mg.mL^−1^, corresponding to half of the IC_50_, was chosen, for according to Vigerelli et al. [11] this dose would cause virus inhibition of about 20 % and neglected cytotoxic effects.

Antiviral synergic effects have already been attributed to amphibian antimicrobial peptides: Chinchar et al. [30] describe a combined effect between magainin II and ranatuerin-2P. However, these authors are unsure whether this would be a synergic effect or a simple additive effect, for they were able to obtain the same level of innactivation by simply doubling the peptide dose. Rollings-Smith et al. [31], on the other hand, were able to describe a true synergic effect between magainin II and PGLa, which present individual MICs of about 100 μM for *Batrachochytrium dendrobatidis*; but, when added together (1:1) to this fungus, their combined MIC drops to 12.5 μM (e.g., 6.25 μM each). Those authors use the argument that antimicrobial peptides are naturally produced and secreted into the skin as a mixture; therefore, synergism should occur. Based on this same principle, the synergism between OF1 and bufotenine could also be expected and, from the amphibian perspective, desirable.

## Conclusions

Our study shows the synergic effect of OF1 and bufotenine against the PV strain of the rabies virus. This is, to the best of these authors’ knowledge, the first description of a synergic antiviral effect between a peptide and an alkaloid. Moreover, the peptide and the alkaloid are not acting on the virus; rather, their effect (individual or synergic) is on the viral penetration into the cell. Further investigations are being conducted in order to evaluate the action mechanism of the antiviral effect, as well as the possible in vivo protective effects of this molecular association.

### Ethics committee approval

The collection and housing of *L. labyrinthicus* were performed under license number 15964–1 from the Brazilian Institute of Environment and Renewable Natural Resources (IBAMA).

## Additional files

Additional file 1:
**Mass spectrometry profile of the active fraction (F11) from** ***L. labyrinthicus*** **skin secretion.** Left black arrows indicate the charge states of the 2547.99 Da molecule and right white arrows indicate the charge states of the 2192.81 Da peptide. (PDF 23 kb) Additional file 2:
**CID MS2 fragmentation spectra of (** **A** **) m/z = 850.16 [M + 3H+]** ^**3+**^ **present in F11 and (** **B** **) the triply charged precursor of synthetic ocellatin-F1 (OF1) (m/z = 850.25).** The most evident peaks are annotated according to the fragmentation pattern. (PDF 34 kb) Additional file 3:
**Figure showing direct immunofluorescence (DIF) of BHK-21 cell monolayer after treatment with RABV and: (** **A** **) 4 mg.mL** ^**−1**^ **synthetic ocellatin-F1 tetrapeptide (OF1TP); (** **B** **) 4 mg.mL** ^**−1**^ **synthetic rabies virus glycoprotein G tetrapeptide (RVGTP); (** **C** **) negative control (MEM-FBS only) and (** **D** **) positive control (PV only).** Magnification 200 × . (PDF 35 kb) Additional file 4:
**Evaluation of cytotoxicity of different molecules in BHK-21 cell line after treatment with: (** **A** **) F11 (1 mg.mL** ^**−1**^ **); (** **B** **) OF1 (1 mg.mL** ^**−1**^ **); (** **C** **) OF1TP (6 mg.mL** ^**−1**^ **); (D) RGVTP (6 mg.mL** ^**−1**^ **) and (** **E** **) bufotenine (0.8 mg.mL** ^**−1**^ **).** (F) Negative control (MEM-FBS only) and (G) positive control [DMSO (1:5) only]. Magnification 100 × . (PDF 89 kb)
