# Supplementary material for: Synergic effects between ocellatin-F1 and bufotenine on the inhibition of BHK-21 cellular infection by the rabies virus
Source: J Venom Anim Toxins Incl Trop Dis. 2015 Dec 2;21:50. doi: 10.1186/s40409-015-0048-1 (PMC4668702; doi:10.1186/s40409-015-0048-1)
Supplement: Additional file 1: — Mass spectrometry profile of the active fraction (F11) from L. labyrinthicus skin secretion. Left black arrows indicate the charge states of the 2547.99 Da molecule and right white arrows indicate the charge states of the 2192.81 Da peptide. (PDF 23 kb) [file 40409_2015_48_MOESM1_ESM.pdf]

Additional file 1

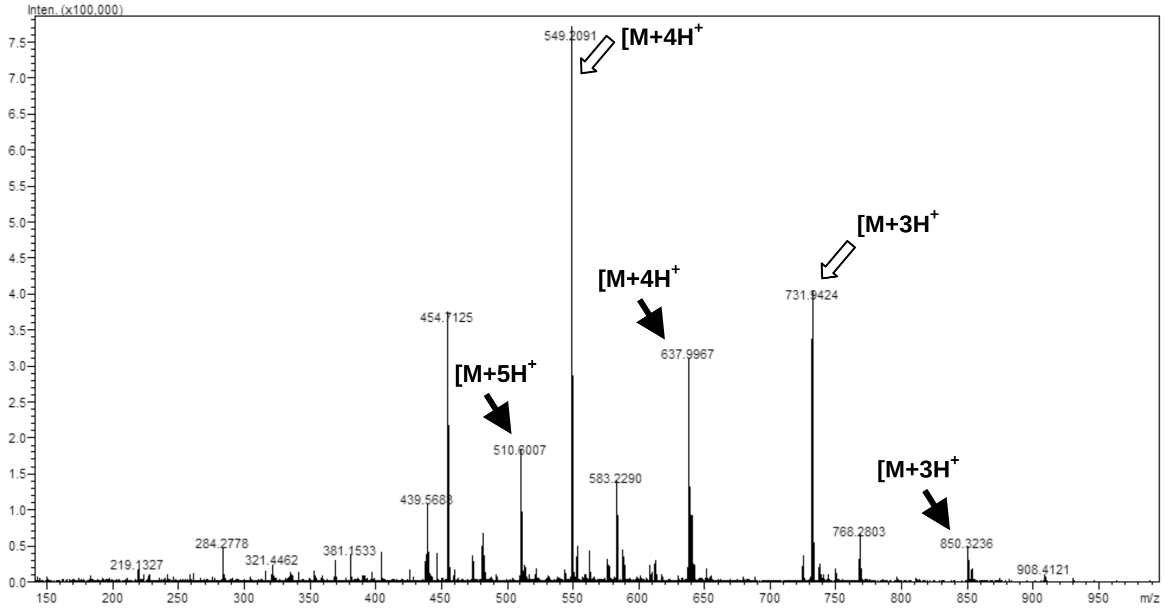

Figure S1. Mass spectrometry profile of the active fraction (F11) of *L. labyrinthicus* skin secretion. Left black arrows indicate the charge states of the 2547.99 Da molecule and right white arrows indicate the charge states the 2192.81 Da peptide.
