# Supplementary material for: Synergic effects between ocellatin-F1 and bufotenine on the inhibition of BHK-21 cellular infection by the rabies virus
Source: J Venom Anim Toxins Incl Trop Dis. 2015 Dec 2;21:50. doi: 10.1186/s40409-015-0048-1 (PMC4668702; doi:10.1186/s40409-015-0048-1)
Supplement: Additional file 2: — CID MS2 fragmentation spectra of ( A ) m/z = 850.16 [M + 3H+] 3+ present in F11 and ( B ) the triply charged precursor of synthetic ocellatin-F1 (OF1) (m/z = 850.25). The most evident peaks are annotated according to the fragmentation pattern. (PDF 34 kb) [file 40409_2015_48_MOESM2_ESM.pdf]

Additional file 2

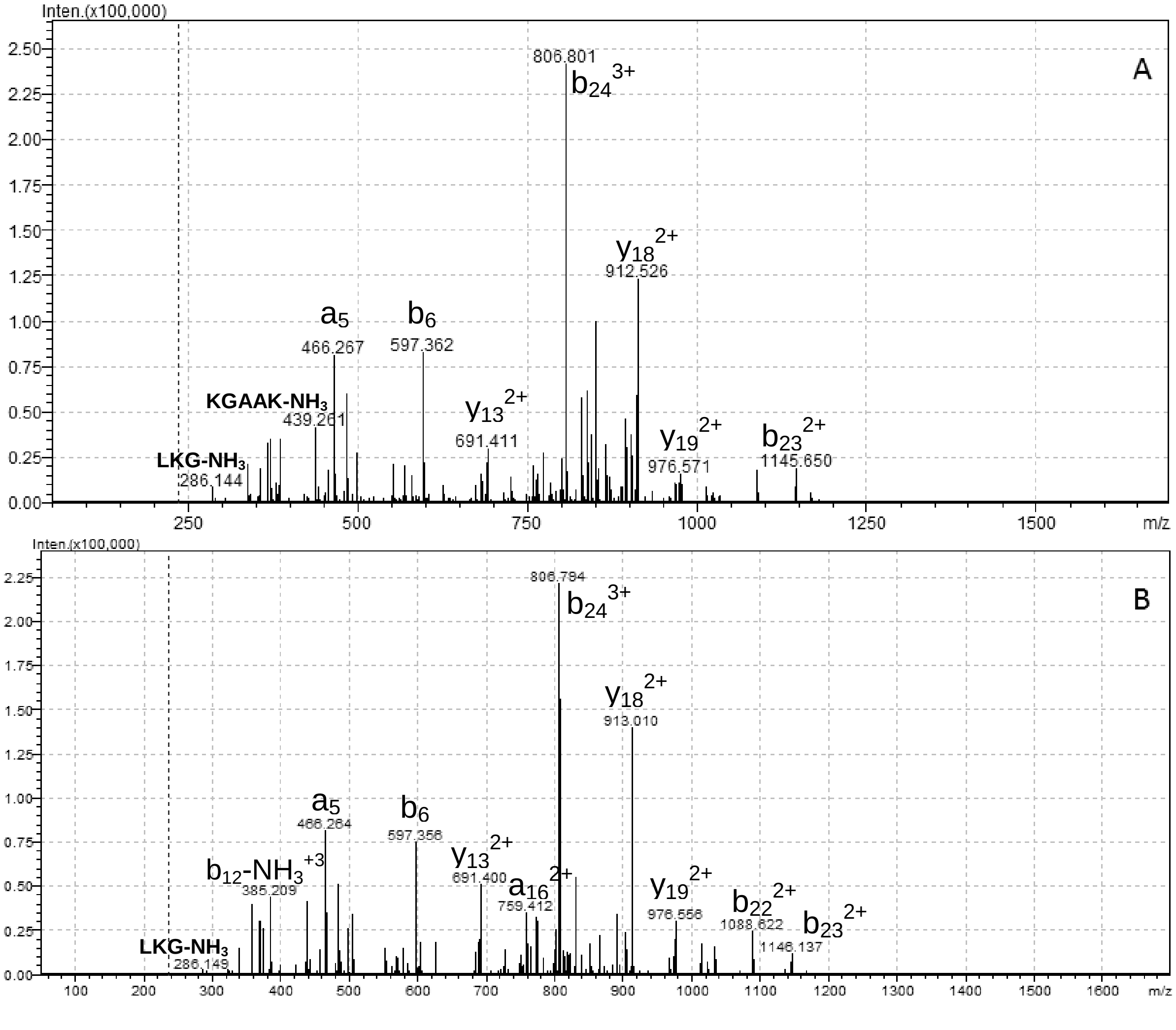

Figure S2. CID MS2 fragmentation spectra of **(A)** m/z=850.16 [M+3H+]3+ present in F11 and **(B)** the triply charged precursor of synthetic ocellatin-F1 (OF1) (m/z=850.25). The most evident peaks are annotated according to the fragmentation pattern.
