# Supplementary material for: Synergic effects between ocellatin-F1 and bufotenine on the inhibition of BHK-21 cellular infection by the rabies virus
Source: J Venom Anim Toxins Incl Trop Dis. 2015 Dec 2;21:50. doi: 10.1186/s40409-015-0048-1 (PMC4668702; doi:10.1186/s40409-015-0048-1)
Supplement: Additional file 3: — Figure showing direct immunofluorescence (DIF) of BHK-21 cell monolayer after treatment with RABV and: ( A ) 4 mg.mL −1 synthetic ocellatin-F1 tetrapeptide (OF1TP); ( B ) 4 mg.mL −1 synthetic rabies virus glycoprotein G tetrapeptide (RVGTP); ( C ) negative control (MEM-FBS only) and ( D ) positive control (PV only). Magnification 200 × . (PDF 35 kb) [file 40409_2015_48_MOESM3_ESM.pdf]

Additional file 3

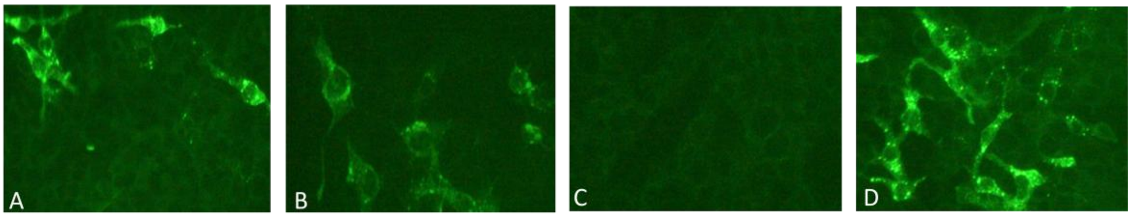

Figure S3. Figure showing direct immunofluorescence (DIF) of BHK-21 cell monolayer after treatment with RABV and: (A) 4 $mg.mL^{-1}$ synthetic ocellatin-F1 tetrapeptide (OF1TP); (B) 4$mg.mL^{-1}$ synthetic rabies virus glycoprotein G tetrapeptide (RVGTP); (C) negative control (MEM-FBS only) and (D) positive control control (PV olny). Magnification 200x.
