# Supplementary material for: Synergic effects between ocellatin-F1 and bufotenine on the inhibition of BHK-21 cellular infection by the rabies virus
Source: J Venom Anim Toxins Incl Trop Dis. 2015 Dec 2;21:50. doi: 10.1186/s40409-015-0048-1 (PMC4668702; doi:10.1186/s40409-015-0048-1)
Supplement: Additional file 4: — Evaluation of cytotoxicity of different molecules in BHK-21 cell line after treatment with: ( A ) F11 (1 mg.mL −1 ); ( B ) OF1 (1 mg.mL −1 ); ( C ) OF1TP (6 mg.mL −1 ); (D) RGVTP (6 mg.mL −1 ) and ( E ) bufotenine (0.8 mg.mL −1 ). (F) Negative control (MEM-FBS only) and (G) positive control [DMSO (1:5) only]. Magnification 100 × . (PDF 89 kb) [file 40409_2015_48_MOESM4_ESM.pdf]

Additional file 4

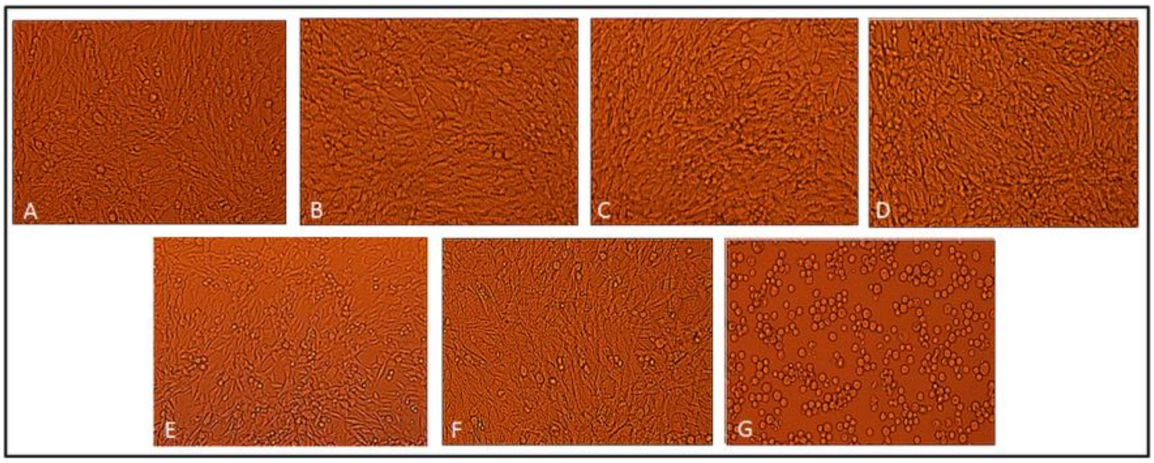

Figure S4. Evaluation of citotoxicity of different molecules in BHK-21 cell line after treatment with: (A) F11 (1 $mg.mL^{-1}$); (B) OF1 (1 $mg.mL^{-1}$); (C) OF1TP (6 $mg.mL^{-1}$); (D) RGVTP (6 $mg.mL^{-1}$) and (E) bufotenine (0.8 $mg.mL^{-1}$). (F) negative control (MEM-FBS only) and (G) positive control (DMSO (1:5) only). Magnification 100x.
